# Peptide-Based Vaccines: Foot-and-Mouth Disease Virus, a Paradigm in Animal Health

**DOI:** 10.3390/vaccines9050477

**Published:** 2021-05-08

**Authors:** Mar Forner, Rodrigo Cañas-Arranz, Sira Defaus, Patricia de León, Miguel Rodríguez-Pulido, Llilianne Ganges, Esther Blanco, Francisco Sobrino, David Andreu

**Affiliations:** 1 Departament de Ciències Experimentals i de la Salut (DCEXS-UPF), 08003 Barcelona, Spain; fornermar@gmail.com (M.F.); sira.defaus@upf.edu (S.D.); 2 Centro de Biología Molecular “Severo Ochoa” (CSIC-UAM), 28049 Madrid, Spain; rcannas@cbm.csic.es (R.C.-A.); pdeleon@cbm.csic.es (P.d.L.); mrrodriguez@cbm.csic.es (M.R.-P.); 3 Centre de Recerca en Sanitat Animal (CReSA), OIE Reference Laboratory for Classical Swine Fever, Institute of Agrifood Research and Technology, 08193 Barcelona, Spain; Llilianne.ganges@irta.cat; 4 Centro de Investigación en Sanidad Animal (CISA-INIA), 28130 Valdeolmos, Spain; blanco@inia.es

**Keywords:** epitope-based vaccines, peptide, vaccines, FMDV, veterinary medicine

## Abstract

Vaccines are considered one of the greatest global health achievements, improving the welfare of society by saving lives and substantially reducing the burden of infectious diseases. However, few vaccines are fully effective, for reasons ranging from intrinsic limitations to more contingent shortcomings related, e.g., to cold chain transport, handling and storage. In this context, subunit vaccines where the essential antigenic traits (but not the entire pathogen) are presented in rationally designed fashion have emerged as an attractive alternative to conventional ones. In particular, this includes the option of fully synthetic peptide vaccines able to mimic well-defined B- and T-cell epitopes from the infectious agent and to induce protection against it. Although, in general, linear peptides have been associated to low immunogenicity and partial protection, there are several strategies to address such issues. In this review, we report the progress towards the development of peptide-based vaccines against foot-and-mouth disease (FMD) a highly transmissible, economically devastating animal disease. Starting from preliminary experiments using single linear B-cell epitopes, recent research has led to more complex and successful second-generation vaccines featuring peptide dendrimers containing multiple copies of B- and T-cell epitopes against FMD virus or classical swine fever virus (CSFV). The usefulness of this strategy to prevent other animal and human diseases is discussed.

## 1. Introduction

**Figure 1:**
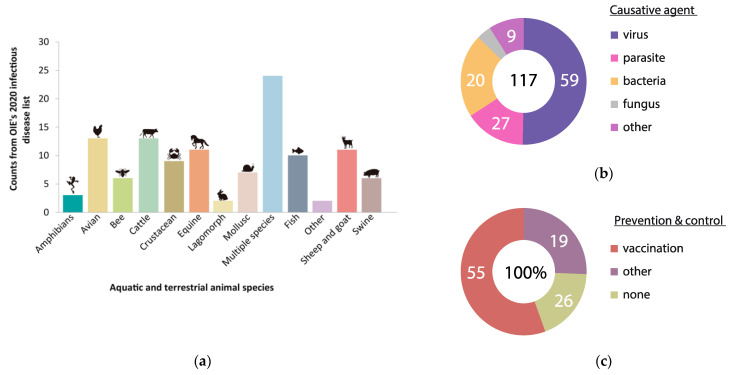
Animal Diseases, Infections and Infestations: (**a**) Counts of terrestrial and aquatic animal diseases published at OIE’s 2020 infectious diseases list; (**b**) Causative agent of the animal diseases reported in absolute values; (**c**) Prevention and control treatments in animal diseases reported in percentage values.

In an era of globalization, the presence of animal disease has a decisive impact on the economic growth prospects of most countries as it affects, beyond the sanitary status of the actual animal populations involved, the viability of many related sectors such as public health, trade, environment and tourism, among others [1]. Importantly, the majority of novel, emergent infectious diseases originate in animals in a manner mainly associated with human activities, which makes animal disease detection and control of paramount interest (One World One Health) [2]. Over the years, the international community has reached the compromise of establishing global, transparent and democratic policies to address such epidemiological issues [3]. As a result, sanitary measures are mainly based on international standards, guidelines and recommendations that are regularly updated and consensually adopted by countries at the annual meetings of the World Organization for Animal Health (also known as the Office International des Epizooties, OIE). Despite these efforts, a real picture of today’s animal health situation lays bare the crucial need for stronger initiatives in veterinary health [4]. According to the 2020 OIE’s Animal Diseases, Infections and Infestations list, there are 117 conditions caused by different pathogenic agents that affect thirteen categories of terrestrial and aquatic animals, which can be largely prevented and controlled by vaccination (Figure 1).

Although nearly 75% of such reported diseases have a preventive strategy, to date only one—rinderpest in cattle and other ruminant animals—has been eradicated. Indeed, the World Health Organization (WHO) has reported that infectious disease is the second major cause of death—after slaughter—in animals. To make sense of these statistics, one should bear in mind that vaccination—arguably the most rational, cost-effective and successful strategy to curb infectious disease, prevent its associated economic losses and increase the lifespan of livestock—is in practice only moderately successful. Few vaccines are fully effective, for diverse reasons ranging from inherent limitations (see below) to more contingent shortcomings related, e.g., to cold chain transport, handling and storage. This review will focus on peptide-based, fully synthetic vaccines that aim to overcome many of the pitfalls of conventional ones. We start with a general survey of epitope-based vaccines and spotlight foot-and-mouth disease virus (FMDV) as a model for peptide-based vaccine development. In addition, advances on the development of analogous peptide vaccines against another important animal virus, namely classical swine fever virus (CSFV), are also reviewed. 

### Current State of FMD Vaccines

FMD vaccines were one of the earliest animal vaccines developed, with first attempts to immunize animals by exposure to infectious virus at the end of the 19th century (for review see [5,6,7,8]). Based on the acquired experience, vaccination has become the predominant instrument to globally control FMD, although no vaccine is able to cross-protect against all serotypes or even between some subtypes of the virus. Current vaccines consist of chemically inactivated (i.e., “killed”) purified whole virus preparations, as vaccination efforts using attenuated virus showed unacceptable danger of virulence reversion in vaccinated animals [9]. To prepare the vaccine, FMDV isolates that antigenically match those circulating in the field should be (i) adapted to grow in an established cell line (e.g., BHK-21) for virus amplification [10,11,12], (ii) inactivated by treatment with binary ethyleneimine (BEI) [13] (iii) purified to remove FMDV nonstructural proteins (NSPs) for serological DIVA (differentiating infected from vaccinated animals) testing, and (iv) formulated with adjuvant/s (aluminum hydroxide or saponin for ruminants and mineral oil adjuvants for other target species). From this perspective, industrial vaccine production requires costly high containment facilities and advanced logistics to constantly adapt vaccine strains and grow large quantities of virulent virus, with the permanent risk of virus escape. Other important limitations include FMD viral particle thermolability and dependency on a cold chain to preserve virus stability and immunogenicity, which increase the manufacturing cost [14].

Inactivated FMD vaccines meet OIE standards and have been effective to control FMD in some regions. However, these vaccines provide short-lived protection (4–6 months) and require revaccination at least every six months, increasing the probabilities of NSPs contamination that leads to false positive results [15,16,17,18]. Additionally, conventional vaccines against FMD do not provide sterile immunity, so vaccinated animals may become persistently infected if exposed to infectious virus, resulting in a carrier state of FMDV [19], which is one of the major threats for livestock international trade. Such concerns led EU and Western countries to endorse severe programs based on test-and-slaughter policies—the so-called “stamping out” procedure—to maintain the “disease-free status” of the country. After multiple FMD outbreaks in non-endemic countries, this policy was translated into wholesale killing of infected and contact animals [20,21], refocusing OIE’s regulation into a new “vaccination-to-live” policy (EU directive 2003/85/EC). This new policy made a clear move to support vaccination campaigns as a measure to counteract FMD, but, also, warned of the need to improve current vaccines [22]. 

## 2. Overview of Epitope-Based Vaccines

Although most vaccines licensed in veterinary medicine use the entire attenuated or inactivated pathogen (Table 1), cells of the adaptive immune system do not recognize it as a whole, but molecular portions of it known as epitopes [23]. In fact, the majority of pathogen proteins are unnecessary for achieving a full protective response and, indeed, some of them can lead to unwanted side effects such as allergy, autoimmunity and off-target responses. These and other safety concerns set the basis for a new generation of vaccines, termed “epitope-based”, consisting of the minimal subset of immunodominant regions responsible for inducing positive, desirable B- and T-cell mediated immune responses [24].

**Figure 2:**
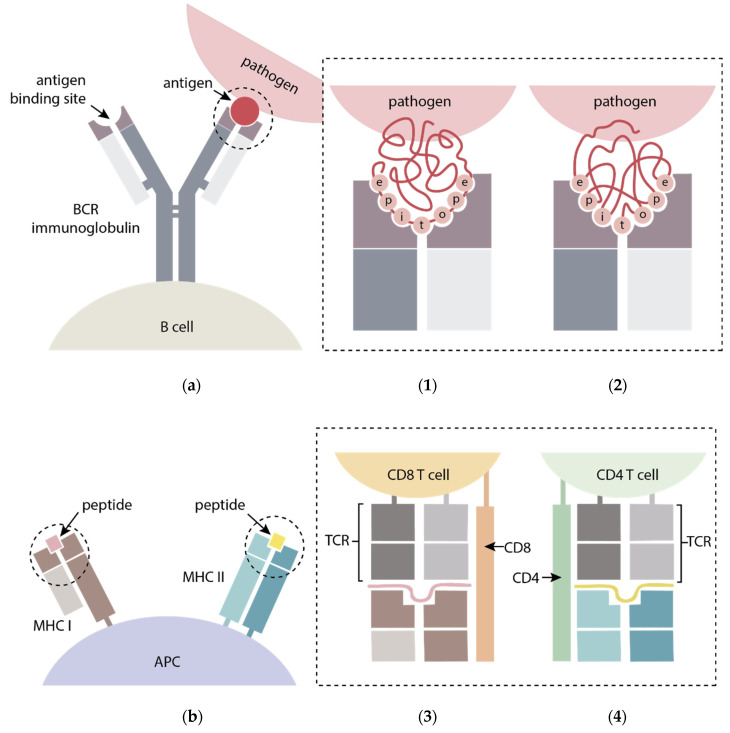
General scheme of (**a**) B-cell epitope recognition through: (**1**) linear (continuous residues); and (**2**) conformational (discontinuous residues) B-cell epitopes from antigens binding B-cell receptor (BCR) immunoglobulins displayed on the surface of/attached to B-cells; (**b**) T-cell epitope recognition by peptides derived from antigens presented via major histocompatibility complex (MHC) I and II molecules bound to APCs and recognized by (**3**) CD8+ and (**4**) CD4+ T-cells T cell receptors (TCR), respectively. Adapted from [28,29].

B-cells generally recognize solvent-exposed motifs and bind, via B-cell receptors (BCRs), antigen epitopes that can be either linear peptide sequences or conformational epitopes, made up by sequence-remote residues brought spatially close by the three-dimensional folding of the antigen (Figure 2a). For their part, T-cells recognize antigens through a T-cell receptor (TCR) when the antigen is loaded on the surface of antigen-presenting cells (APCs) bound to molecules of the major histocompatibility complex (MHC). T-cell epitopes are sequential (continuous) peptides resulting from processed or partially degraded antigens presented by MHC class I (MHC I) or II (MHC II) molecules that interact with two different types, CD8+ and CD4+ T-cells, respectively (Figure 2b) [25,26,27]. The gene polymorphisms in MHC class II and I influenced the recognition of the individual T-cell epitopes. Therefore, it is crucial an adequate selection of T-epitopes able to be recognized by MHC alleles frequently represented in the host population target of vaccination. The formulation of peptide-based vaccines comprising promiscuous T-cell epitopes, ensure an efficient population coverage and a successful vaccine development.

An appropriate identification of immunogenic motifs in antigens is the first fundamental step when designing epitope-based vaccines [30]. Advances in knowledge on B-cell epitope sequence and structure have been achieved by techniques such as nuclear magnetic resonance (NMR), and/or X-ray crystallography of antigen-antibody three-dimensional structure complexes [31,32], and by epitope mapping using predictive bioinformatic algorithms [33] and/or peptide library screening by antibody binding assays [34,35,36]. On the other hand, T-cell epitopes can be predicted by means of bioinformatic algorithms and experimentally characterized using MHC multimers and lymphoproliferation or ELISPOT assays [37,38].

Immunogenic epitopes can be faithfully reproduced by peptides, generated by either genetic engineering or (generally far more accurately) chemical synthesis. In all cases, these approaches imply the absence of infectious agent throughout the vaccine manufacturing processes, hence the avoidance of many risks associated with classical vaccine production [39]. Another desirable feature fulfilled by subunit (e.g., peptide-based) vaccines is that they can be designed to allow differentiating infected from vaccinated animals (DIVA condition) by straightforward serological tests [40]. In addition, peptides in freeze-dried form are chemically stable, even at ambient temperature, thus eliminating the need for constant cold-chain storage and transport required by conventional vaccines [41]. Despite these advantages, the peptide-based approach to vaccines is not free from challenges (e.g., limited immunogenicity of free peptides, intrinsic conformational flexibility that complicates emulating native-like bioactive spatial arrangements, etc.), all of which continue to require special efforts, as discussed hereafter (Table 2). Other related limitations when using a restricted number of viral epitopes as immunogens is that stem from the antigenic heterogeneity of viruses in the field [42]. Such constrains explain why despite many efforts invested in the development of peptide vaccines, only a few related to cancer [43,44,45], malaria [46] and HIV [47] have entered clinical trials.

## 3. FMDV-Specific Immune Response

The development of effective epitope-based vaccines relies on an ever-growing understanding of virus–host interaction and of the mechanisms of immune response against FMDV. Substantial knowledge gaps remain about FMD pathogenesis, but it is reasonably well established that protective immunity, in both animal models and natural hosts, requires the induction of high levels of serotype-specific in vitro neutralizing antibodies (nAbs) [48]. As mentioned above (Section 2), B-lymphocytes recognize pathogen epitopes (in this case on the FMDV particle) to produce specific antibodies that achieve neutralization mainly by preventing FMDV-cell attachment [49,50]. In cattle and pigs, optimal B-cell activation and antibody production relies on the proper activation of components involved in the adaptive mechanism such as Th lymphocytes (also known as T helper cells or CD4+ T lymphocytes) [51]. It is well known that T-cell epitope recognition following antigen processing and presentation in the context of MHC class II molecules stimulates Th lymphocytes to produce cytokines such as IFN-γ, differentiation factors and cognate interactions with B-cells necessary for development of the adaptive immune response against FMDV [52,53].

Additional studies have proposed the phagocytic system as an unconditional companion of effector humoral immunity, by clearance of virus-antibody complexes through in vivo opsonization [49]. Thus, macrophages (MΦ) and dendritic cells (DCs) internalize and degrade FMDV antibody complexes [53,54,55]. Aside from that, DCs are regarded as the most potent antigen-presenting cells (APCs), with a key role in linking the innate with the acquired immune responses [56,57]. The interaction of FMDV with APCs is considered the initial step for developing an effective immune defense that, interestingly, involves both MHC class I and class II molecules [58,59]. Indeed, FMDV infection has been related to significant down-regulation of MHC class I expression on susceptible cells, preventing the presentation of viral peptides by FMDV infected cells to effector cytotoxic T lymphocytes (CTLs) and leading to virus escape [60].

**Figure 3:**
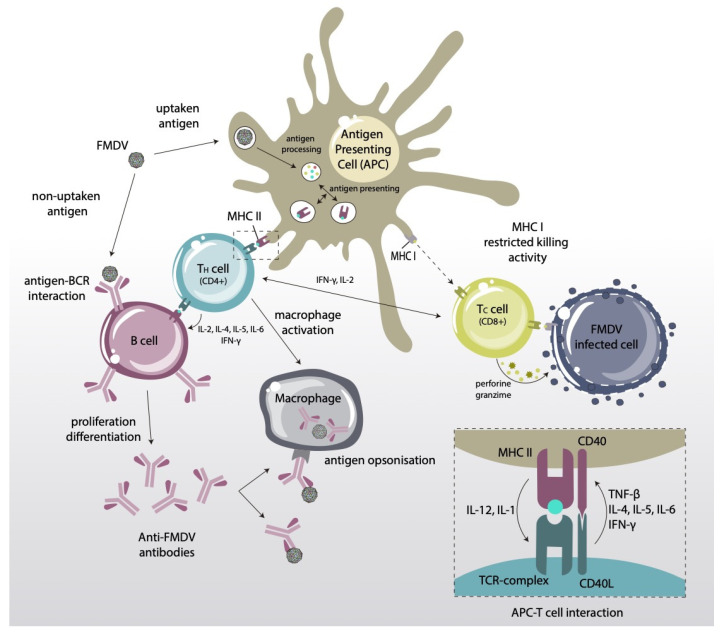
Schematic representation of the adaptive immune response induced by FMDV. APCs present viral peptides (T-cell epitopes) via MHC class I and II molecules to CD8+ and CD4+ T-lymphocytes, respectively. CD4+ T-cells can (i) cooperate with activated B-lymphocytes to produce nAbs; and (ii) interact with CD8+ T-lymphocytes that are previously activated by association with MHC class I molecules on the surface of infected cells, to trigger a cytotoxic (CTL) response.

In certain circumstances, high levels of circulating in vitro nAbs do not correlate with protection while, in contrast, animals with low titers have become protected [49]. These observations would seem to suggest that the effector humoral immunity involves more than high antibody levels, and that the phagocytic system might also play a role in anti-FMDV protection, but the mechanisms involved are poorly understood [61]. An outline of current views on FMDV immune response, including memory effects by activated B- and T-cells, is shown in Figure 3. 

## 4. FMDV Antigenic Structure

### 4.1. B-Cell Epitopes

Over the last few decades, the three-dimensional structures of various FMDV serotypes and subtypes have been solved by X-ray crystallography, enabling a deeper understanding of FMDV antigenicity [62]. Despite their sequence variability, all FMDVs share an icosahedral capsid made up of 60 protomers, each consisting of four structural, VP1–VP4 (VP1–3 external, VP4 internal), plus eleven mature non-structural proteins (NSPs) L_a_, L_ab_, 2A, 2B, 2C, 3A, 3B_1–3_, 3C and 3D, involved in viral replication and interaction with host cells [63,64]. External proteins VP1–3 consist of structurally similar cores containing eight-stranded β-sandwiches of two four-stranded β-sheets connected by surface-exposed loops. Although the entire accessible surface is known to be antigenic, it is in the loops where the main antigenic features have been identified, particularly by using monoclonal antibody (mAb)-resistant (MAR) mutants. Early results on the O_1_ BFS strain evidenced five antigenic sites (1–5): (i) site 1 containing the G–H loop (residues 134 to 158) and the C-terminus (residues 200 to 213) of VP1, (ii) sites 3, 2 and 4 comprising VP1 (residues 43–44, B–C loop), VP2 (residues 70–73, 75, 77 and 131) and VP3 (residue 58) loops, respectively; and (iii) site 5 (residue 149), functionally independent but physically overlapping with site 1 [65]. Similar sites have also been identified in other serotypes and/or subtypes, with the G–H loop as a constant feature [66,67,68]. Aside from linear, continuous site 1 (termed site A in serotype C), with the prominent role in vaccine development discussed below, all other neutralizing sites were conformationally-dependent, discontinuous structures.

In parallel with the above-mentioned studies, enzymatic cleavage of FMDV serotype O corroborated the antigenic role of VP1 and its ability to mediate cell attachment. Thus, trypsin-digested FMDV particles reduced 100-fold the infectivity and 10 to 100-fold the ability to elicit nAbs in guinea pigs [69]. Interestingly, digestion caused VP1 cleavage exclusively at Arg 144 within the G–H loop [70]. This region, structurally disordered and comprising a highly conserved Arg-Gly-Asp (RGD) triplet, serves as a recognition site for host cell integrin receptors [71,72]. Subsequent mapping of VP1 in serotypes O, C and A [73,74,75] using fragments whose immunogenicity had been previously tested in mice, allowed a more accurate definition of immunodominant sites [76,77]. However, vaccination trials using VP1-based immunogens (e.g., recombinant VP1 [78], synthetic B-cell epitope peptides [79], or inclusion at permissive locations within heterologous virus-like particles [80]), showed modest immunogenicity and poor protection, deflating initial expectations. With the benefit of hindsight, such failures are now seen as associated to issues such as a non-native folding of recombinantly made VP1 that occludes antigenic sides or the low amount of epitopes displayed by free (non carrier-conjugated) peptides [81,82]. Additional data on alternative B-cell epitopes outside VP1 have also been described, including continuous immunodominant sites on NSPs [83,84,85].

### 4.2. T-Cell Epitopes

Early studies in natural FMDV hosts allowed the identification of Th epitopes in VP1 [86,87]. Despite the VP1 G-H loop, the high sequence variability of this protein among the multiple FMDV serotypes spurred the search for T-cell epitopes in more conserved domains [88]. Thus, T-cell epitopes for cattle [89] and swine [90] were identified in VP4, a highly conserved structural protein. In particular, peptide VP4(20–34) was shown to bind four different bovine MHC (BoLA) haplotypes and to be presented by MHC class II DQ molecules [91]. The same peptide was also recognized as a porcine T-cell epitope in FMDV-stimulated lymphocytes from vaccinated outbred pigs, providing further evidence of the promiscuous nature of this region [90]. Upon T-cell epitope recognition, effector (inflammatory) or regulatory (suppressive) T-cells can be activated, depending on the co-stimulatory signals expressed [92]. Incorporation of the nucleotide sequence corresponding to the VP4(20–34) epitope into a DNA vaccine turned out to be detrimental in mice, promoting exacerbation of clinical signs after FMDV challenge [93]; and a fusion protein corresponding to VP1(133–158) and VP4(20–34) did not afford full protection in guinea pigs [81]. Thus, further experiments are required to assess the potential of VP4(20–34) as a T helper epitope in natural hosts. 

T-cell epitopes have also been described in NSPs, which generally exhibit low sequence variability among serotypes and are thus likely to be recognized in heterotypic contexts. For instance, overlapping peptides covering all FMDV NSPs allowed identification in pigs of heterotypic and promiscuous T-cell epitopes located in proteins 3A, 3D and 3C [94]. In particular, peptide 3A(21–35) induced lymphoproliferation and cooperated in eliciting nAbs when displayed in linear tandem with the VP1 G-H loop peptide. The potential of this 3A(21–35) T-cell epitope as a key element in novel, fully protective peptide vaccines, has been subsequently confirmed [95,96,97,98] and is described in more detail below.

Other T-cell epitopes have been described in 3D protein in cattle [99] and swine [100]. A recombinant vaccinia virus encoding 3D showed anti-FMDV response, and overlapping peptides spanning 3D allowed the identification of several T-cell epitopes in pigs [91,101]. Similar to 3A(21–35), the inclusion of one of these peptides, 3D(56–70), in a vaccine prototype promoted induction of high nAb titers and a potent IFN-γ response detectable up to 3 months after immunization [102]. As of now, several CD4+ T-cell epitopes identified in cattle and swine are now available as candidates for inclusion into peptide-based vaccines. 

### 4.3. CTL Epitopes

Induction of nAbs and effector T-cells (both responses, not just one) tops the wish list in FMDV vaccine design. In this case, CD8+ T-cells are activated by the recognition of viral-derived peptides associated with MHC class I molecules on the surface of infected cells (Figure 3), to trigger a cytotoxic (CTL) response. Stimulation of CTL responses against exogenously vaccine antigens can be achieved by activation of the so-called cross-presentation pathway. The targeting of T-cell epitopes to dendritic cells (DCs), capable of priming this response, together with the use of toll-like receptors (TLRs) ligands, is a promising strategy for induction of systemic CTL responses. Then, activated CD8+ T-cells directly exert their function by killing virus-infected cells, and indirectly by secreting cytokines that enable the activation of other immune cells. While conventional FMDV vaccines are poor CTL inducers, eliciting CD8+ T-cells against conserved epitopes by vaccination would improve the immune response, especially for highly variable viruses like FMDV [58,103].

Determination of CTL epitopes in outbred populations is a complex task due to the low number of inbred animals within FMDV natural hosts. For this reason, CTL epitope characterization has been mostly addressed by in silico predictions and inbred mice, with the understanding that such models need to be confirmed experimentally. For example, Barfoed et al. reported a murine H2-Kd-restricted CTL epitope that induced cell immunity in mice but did not achieve a protective effect in other animals [104]; in contrast, Gao et al. described several 9-residue peptides from FMDV VP1 that not only induced CTL response in mice but also provided protective response in guinea pigs [105,106,107].

Up to now, there are few reports of FMDV peptides recognized by swine and bovine MHC class I molecules, also referred as swine (SLA) and bovine leukocyte antigen (BoLA), respectively. Guzman et al. described BoLA N*02201-restricted epitopes in VP1(795–803) of FMDV O UKG/2001 inducing a CTL response in vitro by αβ CD8+ but not CD4+ T cells [58]. Other peptides have also been identified by predictive approaches as binding BoLA-Al [108,109] and SLA MHC class I alleles [110,111]. Apart from computational analysis, Ning et al. have studied by X-ray diffraction the interaction between a FMDV CTL epitope and SLA-2*04:02:02, providing the first 3D structure of the peptide binding groove (PBG) and the critical residues that could serve as CTL epitopes [112]. All these studies reveal the potential of FMDV CTL epitopes but the CD8+ response to such peptides remains to be evaluated in vaccinated/infected pigs or cattle.

## 5. Reproducing FMDV Antigenic Sites by Synthetic Peptides

### 5.1. Discontinuous Antigenic Sites

After the characterization of antigenic sites in FMDV, the next step is reproducing them as synthetic peptides that can function as vaccine candidates. This is not an easy task, as most antibodies bind to conformational, discontinuous epitopes [113,114]. In this regard, synthetic peptide replicas of the discontinuous sites must be designed so that they bring epitope-relevant residues spatially close in a native-like arrangement providing adequate epitope-paratope interaction. Furthermore, peptides in solution are far more flexible than folded proteins, so their mobility must somehow be restricted by strategies such as cyclization, which for well-defined structural motifs, e.g., loops, offers some possibilities [115].

**Figure 4:**
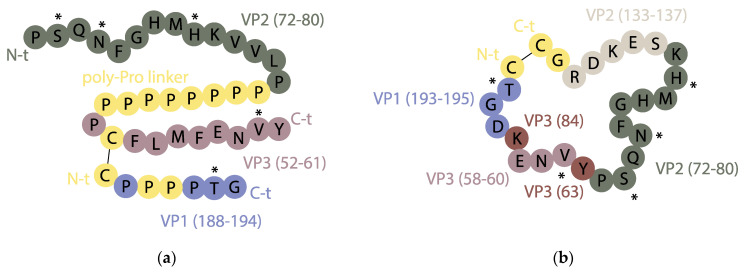
Overview of two synthetic peptides mimicking discontinuous site D of FMDV serotype C using (**a**) a cross-sectional view of the critical residues, with the VP2 and VP3 fragments containing them linked by means of a polyproline helix, and the VP1 segment attached to the VP3 one by a disulfide bond [116]; (**b**) a front view that favors display of surface-exposed antigenic residues [117]. The five critical residues of discontinuous site D are labeled by an asterisk (*) and non-native residues are in yellow. Adapted from [116,117].

An initial approach by our groups was reconstructing discontinuous sites on the basis of the crystal structure of the antigen pinpointing key residues involved in recognition. The goal was to mimic discontinuous antigenic side D of serotype C, which has five critical residues on three external loops of capsid proteins VP1 (residue 193), VP2 (residues 72, 74 and 79) and VP3 (residue 58) recognized by monoclonal Ab resistant (MAR) mutant analysis [114]. These residues and their adjoining regions were combined following the idea of “loop integration”, by means of a disulfide bridge and a poly-proline helix (Figure 4a), into a covalent construct able to elicit modest nAb levels [116]. In a subsequent effort, the cross-sectional view (loop integration) of the capsid was replaced by a “front” side view (surface approach) (Figure 4b), providing a better mimic of the contact surface by displaying only highly exposed elements and avoiding inner residues within the loops [117]. The resulting design was a medium-size disulfide-closed cyclic array able to bind FMDV-derived monoclonal antibodies, thus supporting (by way of NMR studies) the hypothesis that surface-exposed residues were involved in recognition. The designed replica and three analogs defining slightly different ring sizes were inoculated in unconjugated form in guinea pigs, eliciting a FMDV nAb response similar to virus-immunized animals. Unfortunately, vaccination with these peptides did not provide consistent protection against FMDV challenge. To the best of our knowledge, no similar studies attempting to reproduce FMDV discontinuous epitopes have been reported. In any event, some important conclusions could be drawn from that work: (i) cyclic structures above a certain size (ca. 25 residues) may elicit high antibody titers without conjugation to carrier proteins, immune recognition being possibly related to ring size or resistance to proteolysis [118]; (ii) by their reduced flexibility relative to linear counterparts, cyclic peptides can provide reasonable native-like mimicry of conformationally folded, discontinuous viral antigenic sites [119]; (iii) a front-view rather than a cross-sectional approach provides optimized display of relevant, surface-exposed residues involved in antibody recognition [116,117,120,121].

### 5.2. Continuous Antigenic Sites

Although antigenic sites of viral proteins are mainly discontinuous, some sites are indeed continuous, i.e., reproducible by linear peptide replicas, an attractive goal that has stimulated many research efforts. The pioneering work was done on the continuous B-cell epitope at the VP1 G–H loop of FMDV. Linear peptides reproducing this loop from various FMDV serotypes induced nAbs in mice and guinea pigs [122] and achieved limited protection in swine [79]. Further work involved chimeric peptides that juxtaposed two antigenic sites, e.g., the G–H loop and the C-terminus of VP1, in linear fashion, also inducing nAbs [73] and modest protection in swine [123] and cattle [124,125]. Another linear construction, the ACT peptide, integrated site A (G–H loop of VP1), plus the VP1 C-terminus, plus a T-cell epitope [VP1(21–40)] identified in cattle [82,86], again affording partial protection in a large-scale vaccination trial. In a different approach, peptide libraries termed mixotopes [126], reproducing in combinatorial form various antigenically relevant mutations described at the G–H loop were used to immunize guinea pigs [127] with low success: the performance achieved by these linear constructs in host species was significantly lower than that of conventional vaccines [128]. In all those endeavors, the challenge in emulating immunogenicity by the reductionist approach inherent to peptide vaccines was compounded by other issues affecting their development, including among others: (i) the difficulty in correlating neutralizing responses with protection in peptide-vaccinated animals [60,129,130]; (ii) the high degree of antigenic diversity of FMDV; (iii) the lack of recognition of many peptide immunogens by T cells and MHC molecules [89,131]; and (iv) the vulnerability of short peptides to proteases.

Altogether, those early setbacks in achieving protection against FMDV with peptides replicating continuous epitopes helped researchers figure out key elements in engineering more successful candidates. First, the absolute need to include promiscuous T-cell epitopes capable of evoking adequate T-cell responses and optimizing the production of FMDV nAbs [101,132]. Second, the advantages of using more than one epitope copy (single vs. tandem peptides) to circumvent the low immunogenicity of uncoupled peptides [133]. And third, the possibility of minimizing proteolytic degradation by strategies such as cyclization or the use of non-native and/or protease-resistant D-amino acids such as the retroenantio approach [134,135].

## 6. Next-Generation Peptide Vaccines: Multimerization Approaches

As discussed above, linear peptides are poorly immunogenic when administered alone. Considering that in natural contexts, infectious agents usually display several different antigenic motifs, each of them in more than one copy, it seems quite reasonable to attempt to present, in man-made replicas, such epitopes in multimeric fashion, as molecular structures of higher order than individual peptides. This idea has given rise to a broad range of scaffolds where multiple peptide replicas are covalently attached. Examples include template-assisted synthetic protein (TASP) platforms [136], gold nanoparticles (GNP) [137], aromatic hydrocarbons [138], or four-armed star polymers [139], in addition to the classic poly lysine core, multiple antigenic peptide (MAP) system pioneered by Tam [140,141].

**Figure 5:**
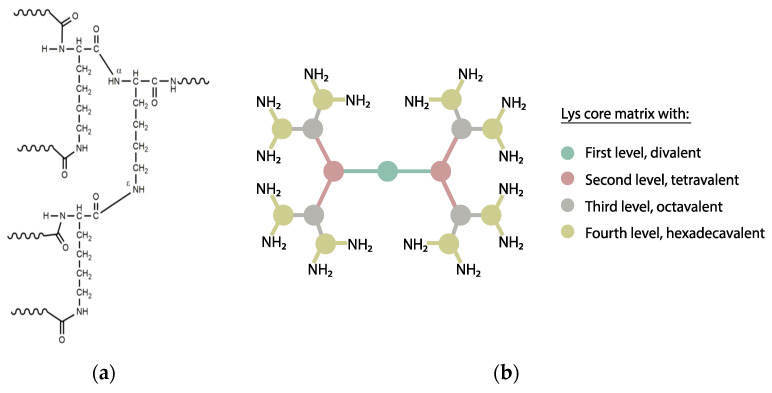
(**a**) Branching N^α^ and N^ε^ amino groups forming the Lys core of a MAP construct; (**b**) Different dendrimer multiplicities starting from a Lys core. Each circle represents a Lys residue, each line a peptide sequence and the colors denote the level of multiplicity.

Tam’s strategy consists of a branched architecture where the peptide motifs grow from the N^α^ and N^ε^ amino groups of a lysine core, giving rise to multivalent, molecularly defined constructs termed dendrimers (Figure 5). From the outset, MAPs have been successfully applied for vaccine purposes [142,143]. Other advantages of MAP immunogens include their (relative) simple design and synthesis, the versatility for achieving combined (e.g., B-and T-cell epitope) immune response, or moderate enzymatic resistance [144].

Preparation of peptide dendrimers involves either standard SPPS methods or conjugation in solution of previously prepared building blocks through various ligation chemistries, e.g., thioether, hydrazine, oxime, thiazolidine, thio-maleimide or azide–alkyne. Thiol-based ligations exploit thiol chemoselectivity for either thiol-disulfide exchanges, or for nucleophilic substitution on alkyl halides (particularly if activated, e.g., α-halocarbonyl), or for Michael-type addition to conjugated olefins. High multiplicity MAPs, i.e., tetra-, octameric or above, pose increased challenges due to steric hindrance (slowing reactivity) and insolubility (intra- and intermolecular aggregation phenomena) [145,146,147]. Alternative methods to address such issues and reduce byproduct formation have been reported, including a reverse thioether ligation route, where a chloroacetyl-derivatized epitope is tethered to a thiol-functionalized form of a Lys dendron core [148].

## 7. FMDV Dendrimer Peptide Vaccines

Multimerization via dendrimer synthesis opens the possibility of displaying well-defined FMDV B-and T-cell epitopes into a single molecule, overcoming the limitations of single peptides. In accordance with this strategy, a tetravalent prototype, hereafter termed B_4_T, was designed and synthesized by our groups. First, a peptide reproducing the heterotypic and highly conserved FMDV 3A(21–35) T-cell epitope was built on solid phase and elongated at the N-terminus by two Lys residues defining a putative cathepsin D cleavage site, and followed by a four-branched Lys core derivatized by chloroacetyl groups. This dendrimer precursor was cleaved from the resin, purified and characterized. In parallel, a B-cell epitope peptide from type C FMDV [VP1 (136–154)] acetylated at the N-terminus and C-terminally elongated with a Cys residue was made and purified. Conjugation of the two components by thioether ligation at pH~7 yielded a crude with B_4_T as the main product. Subsequent HPLC purification allowed to isolate B_4_T-rich fractions that were used for immunization. Two doses of B_4_T elicited high titers of FMDV-nAbs, activated T-cells (IFN-γ release) and induced full protection in swine upon infection with homologous FMDV. The tetrameric construct generated high levels of mucosal IgA preventing virus transmission from challenged to contact control animals [97,149]. Based on these results, the presentation of B-cell epitopes in dendrimeric fashion was found to be essential for protection, as linear versions of the B-cell in tandem with the same T-cell epitope did not protect pigs [96]. In a similar trial on cattle, a B_4_T-like construct afforded also protection, albeit partially [150,151].

**Figure 6:**
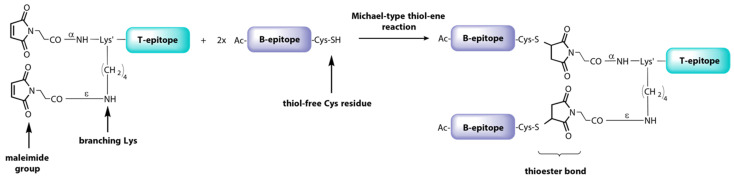
Synthetic scheme of the B_2_T vaccine prototype against FMDV.

These results have stimulated extensive research on dendrimer vaccine prototypes targeted at more epidemiologically relevant FMDV serotypes. Interestingly, a downsized version, named B_2_T, bearing two copies of the B-cell epitope from type O FMDV VP1 protein [VP1 (140–160)] linked to one copy of 3A(21–35) T-cell epitope by means of a much more efficient thiol-maleimide ligation (Figure 6), improved nAb and IFN-γ responses over the tetravalent dendrimer in Swiss CD1 mice [98]. This somehow counterintuitive outcome, where less B-epitope copies elicit better responses, was remarkably validated in pigs, showing full protection (100%, 6/6 animals) against FMDV challenge [95]. In addition, the maleimide-linked B_2_T platform performed also better than an equivalent bivalent but thioether-linked construct, underlining the impact of even the slightest structural details on complex biological events such as vaccination with peptides. Further experiments in swine demonstrated that one single dose of B_2_T was able to elicit high nAb titers against FMDV and activation of IFN-γ-producing cells [152]. Remarkably, long-term anti-FMDV protective responses have been detected up to five months post-immunization even with a 4-fold lower dose, highlighting the feasibility of these dendrimers as FMDV vaccines [102]. 

**Figure 7:**
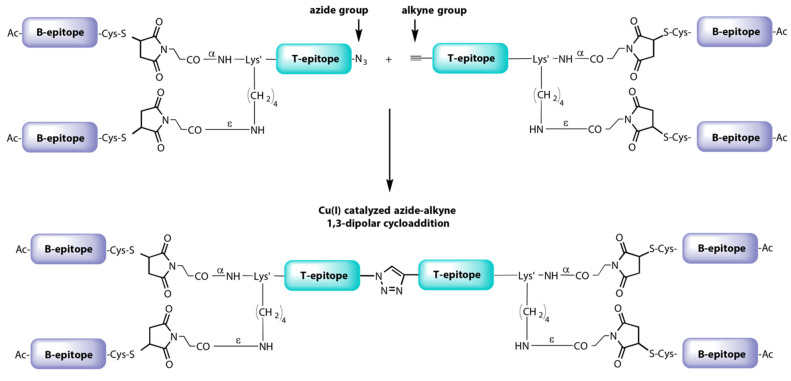
Synthetic scheme of B_2_T-TB_2_ vaccine prototype against FMDV.

More recently, a new dendrimer peptide, named B_2_T-TB_2_, consisting of two B_2_T molecules joined tail-to-tail by click [copper Cu(I)-assisted alkyne-azide cycloaddition] chemistry has been described [147] (Figure 7). The B_2_T-TB_2_ peptide was able to elicit high nAb titers in both mice and swine [153].

The modular design of B_2_T constructs makes replacement of either B- or T-cell epitopes a feasible option. In the latter case, this allows to explore in depth the modulation of the B-cell response by T-cell epitopes. In this line, a B_2_T peptide harboring another T-cell peptide, i.e., that located at 3D(56–70), showed similar nAb levels than those afforded by 3A(21–35), including as well a potent IFN-γ response upon in vitro recall with the homologous dendrimer [154], thereby extending the repertoire of swine T-cell epitopes usable.

The immune response was influenced not only by the presence but also by the orientation of the T-cell epitope [149]. Thus, B_2_TT constructs displaying the T-3A and T-3D epitopes in tandem showed different immunomodulatory effects for the two juxtapositions. Also, the requirement of T-cell epitope inclusion in the peptide vaccine was again confirmed, by comparison with ineffective immunizations with construct B_2_, lacking a T-cell epitope. Our studies also suggest that T-cells producing IFN-γ in response to in vitro recall with the FMDV dendrimer are memory cells (CD4+2E3- T-cells) and, to a lesser extent, cytotoxic T-cells (CD8β+) [155]. 

On the other hand, we have recently addressed the influence of SLA allele composition on the response of 63 pigs to B_2_T constructs [156]. A robust significant correlation between SLA-II Lr-Hp and T-cell response, as well as a slightly less significant correlation to the neutralizing antibody response evoked by the B_2_T dendrimers were found. In particular, allele groups SLA-I Lr-Hp Lr-59.0 and Lr-22.0 as well as Lr-0.15b were found associated with high T-cell responses and nAb titers, respectively. These 3 allele groups are highly abundant Lr-Hp in European farmed pigs, which is of potential value for peptide vaccine design.

All in all, B_2_T-type dendrimers constitute efficient FMDV immunogens, eliciting robust immune responses at both serum and mucosal levels, thus providing a reliable pharmaceutical-like alternative to undefined, risk-prone biologicals such as inactivated vaccines. 

## 8. Extending the Dendrimer Vaccine Approach to Classical Swine Fever

The versatile and customizable nature of B_2_T dendrimers has also been exploited to design vaccines against classical swine fever virus (CSFV), another important pig disease [157]. The immunogenicity against CSFV of three B_2_T-type candidates displaying respectively three different B-cell epitopes from glycoprotein E2 (i.e., (694–712), (712–727) [158], or (829–842)) [159], joined to a T-cell epitope from NS3 protein (residues 1446–1460) was tested in domestic pigs [160,161]. Those B-cell epitopes had been already reported to induce CSFV-neutralizing antibodies when administered in monomeric form [158,162,163,164,165]. However, only pigs immunized with the construct including E2(694–712) B-cell epitope had a high and uniform antibody response against the immunogen, giving rise to partial protection against CSFV challenge [161]. Surprisingly, in another study, a B_2_T prototype combining CSFV B-cell epitopes with FMDV 3A(21–35) T-cell epitope elicited an improved antibody response against CSFV and higher levels of protection against virus challenge than those afforded by the same dendrimer harboring the CSFV T-cell epitope from NS3 protein [166].

In summary, the MAP-inspired B_2_T (or, more generally, B_n_T) strategy stands out for its ability to increase peptide immunogenicity, particularly in swine [97,160,161,167]. The branching arrangement may protect certain residues from enzymatic cleavage, causing epitopes to be processed and presented more efficiently by antigen-presenting cells [167,168]. Altogether, these results support the relevance of dendrimer peptides as an effective way to combine different B- and T-cell epitopes into a single molecular entity with good prospects of vaccine application, plus the ability to explore mechanisms involved in the modulation of the immune response (reviewed in [144]).

## 9. Concluding Remarks

Peptide-based vaccines are attractive and timely alternatives to traditional vaccines [169]. Strategies to improve their low immunogenicity have given rise to plausible candidates against human infectious diseases and cancer, some of them at advanced stages of clinical trials [170,171]. Veterinary medicine must obviously join the trend of novel safer, versatile vaccines to overcome the shortcomings of conventional ones.

For a rational design of an efficient peptide vaccine against viruses—extendable to other pathogens—, three main limitations must be reckoned with. First, the partial knowledge of immune effector mechanisms, especially the role of CD4+ and CD8+ lymphocyte responses in protection. Second, the difficulties in reproducing conformation-dependent B-cell antigenic sites must be kept in mind and addressed, along with conformation-independent (continuous) determinants, which can be easily mimicked by linear peptides and included in synthetic constructs. And third, the intrinsic handicaps of single linear peptides such as their low immunogenicity and short half-life in serum due to enzymatic degradation. Interestingly, peptides facing similar challenges have already shown to be effective against other infectious or genetic diseases [44,170,172,173], so their value in immune protection should not be underestimated. Among the most valuable advantages of peptide vaccines are (i) their inherent safety; (ii) the ability to achieve high antigen density in a single molecule and to avoid antigens with detrimental responses; and (iii) the possibilities of engineering by multimerization, cyclization, etc. 

FMDV peptide vaccines discussed in this review exemplify the steps involved in effective vaccine development, and in particular how various shortcomings can be overcome. Substantial efforts to understand FMDV-host interactions have provided useful clues towards rational design of candidates. However, the genetic diversity of FMDV, common to RNA viruses and translating into good adaptability and, more pertinently, high antigenic variability has limited the odds of a universal subunit (or for that matter conventional) vaccine. 

Multimeric arrangements of well-defined FMDV B- and T-cell epitopes into dendrimer constructs have given rise to novel formulations eliciting robust immune responses in natural host that significantly expand the scope of subunit vaccines. While issues such as dosage, cost-effectiveness, immunization schedules or duration of the protection have been only partially addressed, FMDV dendrimer peptide vaccines stand now as effective candidates likely to enter the veterinary vaccine pipeline in coming years.

Extension of the modular approach underlying dendrimeric vaccines is conceptually possible provided B- and T-cell epitopes capable of evoking protective responses are known. This is the case of CSFV, another important livestock viral disease, for which dendrimer peptides analogous to those developed for FMDV have been shown to confer partial protection against viral challenge. It is noteworthy that the dendrimer approach could also be extended to newly emerging pathogens such as the novel pandemic beta-coronavirus SARS-CoV-2. Indeed, experiments in our laboratories are in progress to assess whether B_2_T-like platforms tailored to SARS-CoV-2 may induce neutralizing nAbs, opening possibilities of contributing to prevent the current pandemic. 

## Figures and Tables

**Table 1:** Vaccine world production values compiled from World Animal Health Information System (WAHIS) interface in 2005 and 2019.

Type of Vaccine	2005	2019
Live attenuated vaccine	402	444
Inactivated vaccine	285	329
Conjugated vaccine	6	6
Recombinant vector vaccine	6	13
Subunit vaccine	1	4
DNA vaccine	-	2

**Table 2:** Pros and cons of peptide-based vaccines.

Pros	Cons
Absence of infectious agent	Laborious identification of antigenic epitopes
No risk of mutation or reversion	Emulating 3D structures
Chemical stability	T-cell epitopes insufficiently specified
DIVA capability	Low immunogenicity
Simple handling, storage and transport	
Easy to manufacture	

## References

[1] Saker L., Lee K., Cannito B., Gilmore A., Campbell-Lendrum D.H. (2004). Globalization and Infectious Diseases: A Review of the Linkages.

[2] Mackenzie J.S., Jeggo M. (2019). The One Health Approach-Why Is It So Important?. Trop. Med. Infect. Dis..

[3] Bonbon E. (2020). A framework for national official assurance systems with reference to World Organisation for Animal Health standards. Rev. Sci. Tech..

[4] Caceres P., Tizzani P., Ntsama F., Mora R. (2020). The World Organisation for Animal Health: Notification of animal diseases. Rev. Sci. Tech..

[5] Diaz-San Segundo F., Medina G.N., Stenfeldt C., Arzt J., de Los Santos T. (2017). Foot-and-mouth disease vaccines. Vet. Microbiol..

[6] Lombard M., Füssel A.E. (2007). Antigen and vaccine banks: Technical requirements and the role of the european antigen bank in emergency foot and mouth disease vaccination. Rev. Sci. Tech..

[7] Blanco E., Andreu D., Sobrino F., Sobrino F., Domingo E. (2017). Peptide Vaccines Against Foot-and-mouth Disease. Foot-and-Mouth Disease Virus. Current Research and Emerging Trends.

[8] de Los Santos T., Diaz-San Segundo F., Rodriguez L.L. (2018). The need for improved vaccines against foot-and-mouth disease. Curr. Opin. Virol..

[9] Doel T.R. (2003). FMD vaccines. Virus Res..

[10] Capstick P.B., Garland A.J., Masters R.C., Chapman W.G. (1966). Some functional and morphological alterations occurring during and after the adaptation of BHK 21 clone 13 cells to suspension culture. Exp. Cell Res..

[11] Capstick P.B., Telling R.C., Chapman W.G., Stewart D.L. (1962). Growth of a cloned strain of hamster kidney cells in suspended cultures and their susceptibility to the virus of foot-and-mouth disease. Nature.

[12] Mowat G.N., Brooksby J.B., Pay T.W. (1962). Use of BHK 21 cells in the preparation of mouse attenuated live foot-and-mouth disease vaccines for the immunization of cattle. Nature.

[13] Bahnemann H.G. (1972). The inactivation of foot-and-mouth disease virus by ethylenimine and propylenimine. Zent. Vet. Reihe B.

[14] Cao Y., Lu Z., Liu Z. (2016). Foot-and-mouth disease vaccines: Progress and problems. Expert Rev. Vaccines.

[15] Barteling S.J., Sobrino F., Domingo E. (2004). Modern inactivated Foot-and-mouth disease (FMD) vaccines: Historical background and key elements in production and use. Foot-and-Mouth Disease: Current Perspectives.

[16] Cox S.J., Aggarwal N., Statham R.J., Barnett P.V. (2003). Longevity of antibody and cytokine responses following vaccination with high potency emergency FMD vaccines. Vaccine.

[17] Parida S. (2009). Vaccination against foot-and-mouth disease virus: Strategies and effectiveness. Expert Rev. Vaccines.

[18] Rodriguez L.L., Gay C.G. (2011). Development of vaccines toward the global control and eradication of foot-and-mouth disease. Expert Rev. Vaccines.

[19] Alexandersen S., Zhang Z., Donaldson A.I. (2002). Aspects of the persistence of foot-and-mouth disease virus in animals--the carrier problem. Microbes Infect..

[20] Kitching P., Hammond J., Jeggo M., Charleston B., Paton D., Rodriguez L., Heckert R. (2007). Global FMD control—Is it an option?. Vaccine.

[21] Sobrino F., Domingo E. (2001). Foot-and-mouth disease in Europe. FMD is economically the most important disease of farm animals. Its re-emergence in Europe is likely to have consequences that go beyond severe alterations of livestock production and trade. EMBO Rep..

[22] Geale D.W., Barnett P.V., Clarke G.W., Davis J., Kasari T.R. (2015). A Review of OIE Country Status Recovery Using Vaccinate-to-Live Versus Vaccinate-to-Die Foot-and-Mouth Disease Response Policies II: Waiting Periods After Emergency Vaccination in FMD Free Countries. Transbound. Emerg. Dis..

[23] Ritmahan W., Kesmir C., Vroomans R.M.A. (2020). Revealing factors determining immunodominant responses against dominant epitopes. Immunogenetics.

[24] Kwong P.D., DeKosky B.J., Ulmer J.B. (2020). Antibody-guided structure-based vaccines. Semin. Immunol..

[25] Paul W.E. (2013). Fundamental Immunology.

[26] Stern L.J., Wiley D.C. (1994). Antigenic peptide binding by class I and class II histocompatibility proteins. Structure.

[27] Van Regenmortel M.H. (2009). What is a B-cell epitope?. Methods Mol. Biol..

[28] Sanchez-Trincado J.L., Gomez-Perosanz M., Reche P.A. (2017). Fundamentals and Methods for T- and B-Cell Epitope Prediction. J. Immunol. Res..

[29] Sette A., Fikes J. (2003). Epitope-based vaccines: An update on epitope identification, vaccine design and delivery. Curr. Opin. Immunol..

[30] Gershoni J.M., Roitburd-Berman A., Siman-Tov D.D., Tarnovitski Freund N., Weiss Y. (2007). Epitope mapping: The first step in developing epitope-based vaccines. Biodrugs Clin. Immunother. Biopharm. Gene Ther..

[31] Benjamin D.C. (1991). Molecular approaches to the study of B cell epitopes. Int. Rev. Immunol..

[32] Cason J. (1994). Strategies for mapping and imitating viral B-cell epitopes. J. Virol. Methods.

[33] Potocnakova L., Bhide M., Pulzova L.B. (2016). An Introduction to B-Cell Epitope Mapping and In Silico Epitope Prediction. J. Immunol. Res..

[34] Di Marco M., Peper J.K., Rammensee H.G. (2017). Identification of Immunogenic Epitopes by MS/MS. Cancer J..

[35] Pinilla C., Appel J.R., Judkowski V., Houghten R.A. (2012). Identification of B cell and T cell epitopes using synthetic peptide combinatorial libraries. Curr. Protoc. Immunol..

[36] Vanniasinkam T., Barton M.D., Das T.P., Heuzenroeder M.W. (2018). B-Cell Epitope Mapping Using a Library of Overlapping Synthetic Peptides in an Enzyme-Linked Immunosorbent Assay. Methods Mol. Biol..

[37] Rosa D.S., Ribeiro S.P., Cunha-Neto E. (2010). CD4+ T cell epitope discovery and rational vaccine design. Arch. Immunol. Ther. Exp..

[38] Sidney J., Peters B., Sette A. (2020). Epitope prediction and identification- adaptive T cell responses in humans. Semin. Immunol..

[39] Patarroyo M.E., Patarroyo M.A. (2008). Emerging rules for subunit-based, multiantigenic, multistage chemically synthesized vaccines. Acc. Chem. Res..

[40] Uttenthal A., Parida S., Rasmussen T.B., Paton D.J., Haas B., Dundon W.G. (2010). Strategies for differentiating infection in vaccinated animals (DIVA) for foot-and-mouth disease, classical swine fever and avian influenza. Expert Rev. Vaccines.

[41] Matthias D.M., Robertson J., Garrison M.M., Newland S., Nelson C. (2007). Freezing temperatures in the vaccine cold chain: A systematic literature review. Vaccine.

[42] Domingo E. (1992). Genetic variation and quasi-species. Curr. Opin. Genet. Dev..

[43] Kyte J.A., Gaudernack G., Dueland S., Trachsel S., Julsrud L., Aamdal S. (2011). Telomerase peptide vaccination combined with temozolomide: A clinical trial in stage IV melanoma patients. Clin. Cancer Res..

[44] Lennerz V., Gross S., Gallerani E., Sessa C., Mach N., Boehm S., Hess D., von Boehmer L., Knuth A., Ochsenbein A.F. (2014). Immunologic response to the survivin-derived multi-epitope vaccine EMD640744 in patients with advanced solid tumors. Cancer Immunol. Immunother..

[45] Zhang L. (2018). Multi-epitope vaccines: A promising strategy against tumors and viral infections. Cell. Mol. Immunol..

[46] Tsuji M., Zavala F. (2001). Peptide-based subunit vaccines against pre-erythrocytic stages of malaria parasites. Mol. Immunol..

[47] Kunwar P., Hawkins N., Dinges W.L., Liu Y., Gabriel E.E., Swan D.A., Stevens C.E., Maenza J., Collier A.C., Mullins J.I. (2013). Superior control of HIV-1 replication by CD8+ T cells targeting conserved epitopes: Implications for HIV vaccine design. PLoS ONE.

[48] McCullough K.C., Sobrino F., Sobrino F., Domingo E. (2004). Immunology of Foot-and-mouth disease. Foot-and-Mouth Disease: Current Perspective.

[49] McCullough K.C., De Simone F., Brocchi E., Capucci L., Crowther J.R., Kihm U. (1992). Protective immune response against foot-and-mouth disease. J. Virol..

[50] McCullough K.C., Summerfield A. (2005). Basic concepts of immune response and defense development. ILAR J..

[51] McCullough K.C., Pullen L., Parkinson D. (1992). The immune response against foot-and-mouth disease virus: Influence of the T lymphocyte growth factors IL-1 and IL-2 on the murine humoral response in vivo. Immunol. Lett..

[52] Halabi G., McCullough K.C. (1995). Influence of antigen presentation and exogenous cytokine activity during in vitro primary immunizations employed for the generation of monoclonal antibodies. J. Immunol. Methods.

[53] McCullough K.C., Parkinson D., Crowther J.R. (1988). Opsonization-enhanced phagocytosis of foot-and-mouth disease virus. Immunology.

[54] Baxt B., Mason P.W. (1995). Foot-and-mouth disease virus undergoes restricted replication in macrophage cell cultures following Fc receptor-mediated adsorption. Virology.

[55] McCullough K.C., Crowther J.R., Butcher R.N., Carpenter W.C., Brocchi E., Capucci L., De Simone F. (1986). Immune protection against foot-and-mouth disease virus studied using virus-neutralizing and non-neutralizing concentrations of monoclonal antibodies. Immunology.

[56] McCullough K.C., Ruggli N., Summerfield A. (2009). Dendritic cells—At the front-line of pathogen attack. Vet. Immunol. Immunopathol..

[57] Summerfield A., McCullough K.C. (2009). The porcine dendritic cell family. Dev. Comp. Immunol..

[58] Guzman E., Taylor G., Charleston B., Ellis S.A. (2010). Induction of a cross-reactive CD8(+) T cell response following foot-and-mouth disease virus vaccination. J. Virol..

[59] Sáiz J.C., Rodríguez A., González M., Alonso F., Sobrino F. (1992). Heterotypic lymphoproliferative response in pigs vaccinated with foot-and-mouth disease virus. Involvement of isolated capsid proteins. J. Gen. Virol..

[60] Sobrino F., Sáiz M., Jiménez-Clavero M.A., Núñez J.I., Rosas M.F., Baranowski E., Ley V. (2001). Foot-and-mouth disease virus: A long known virus, but a current threat. Vet. Res..

[61] Summerfield A., Guzylack-Piriou L., Harwood L., McCullough K.C. (2009). Innate immune responses against foot-and-mouth disease virus: Current understanding and future directions. Vet. Immunol. Immunopathol..

[62] Fry E.E., Stuart D.I., Rowlands D.J. (2005). The structure of foot-and-mouth disease virus. Curr. Top. Microbiol. Immunol..

[63] Liu W., Shao J., Chen D., Chang Y., Chang H., Zhang Y. (2019). Identification of three linear B cell epitopes against non-structural protein 3ABC of FMDV using monoclonal antibodies. Appl. Microbiol. Biotechnol..

[64] Jaworski J.P., Compaired D., Trotta M., Perez M., Trono K., Fondevila N. (2011). Validation of an r3AB1-FMDV-NSP ELISA to distinguish between cattle infected and vaccinated with foot-and-mouth disease virus. J. Virol. Methods.

[65] Asfor A.S., Upadhyaya S., Knowles N.J., King D.P., Paton D.J., Mahapatra M. (2014). Novel antibody binding determinants on the capsid surface of serotype O foot-and-mouth disease virus. J. Gen. Virol..

[66] Acharya R., Fry E., Stuart D., Fox G., Rowlands D., Brown F. (1989). The three-dimensional structure of foot-and-mouth disease virus at 2.9 A resolution. Nature.

[67] Curry S., Fry E., Blakemore W., Abu-Ghazaleh R., Jackson T., King A., Lea S., Newman J., Rowlands D., Stuart D. (1996). Perturbations in the surface structure of A22 Iraq foot-and-mouth disease virus accompanying coupled changes in host cell specificity and antigenicity. Structure.

[68] Lea S., Hernández J., Blakemore W., Brocchi E., Curry S., Domingo E., Fry E., Abu-Ghazaleh R., King A., Newman J. (1994). The structure and antigenicity of a type C foot-and-mouth disease virus. Structure.

[69] Wild T.F., Burroughs J.N., Brown F. (1969). Surface structure of foot-and-mouth disease virus. J. Gen. Virol..

[70] Niepmann M. (1996). Porcine polypyrimidine tract-binding protein stimulates translation initiation at the internal ribosome entry site of foot-and-mouth-disease virus. FEBS Lett..

[71] Fox G., Parry N.R., Barnett P.V., McGinn B., Rowlands D.J., Brown F. (1989). The cell attachment site on foot-and-mouth disease virus includes the amino acid sequence RGD (arginine-glycine-aspartic acid). J. Gen. Virol..

[72] Mason P.W., Piccone M.E., McKenna T.S., Chinsangaram J., Grubman M.J. (1997). Evaluation of a live-attenuated foot-and-mouth disease virus as a vaccine candidate. Virology.

[73] Strohmaier K., Franze R., Adam K.H. (1982). Location and characterization of the antigenic portion of the FMDV immunizing protein. J. Gen. Virol..

[74] Rowlands D.J., Clarke B.E., Carroll A.R., Brown F., Nicholson B.H., Bittle J.L., Houghten R.A., Lerner R.A. (1983). Chemical basis of antigenic variation in foot-and-mouth disease virus. Nature.

[75] Thomas A.A., Woortmeijer R.J., Puijk W., Barteling S.J. (1988). Antigenic sites on foot-and-mouth disease virus type A10. J. Virol..

[76] Parry N., Fox G., Rowlands D., Brown F., Fry E., Acharya R., Logan D., Stuart D. (1990). Structural and serological evidence for a novel mechanism of antigenic variation in foot-and-mouth disease virus. Nature.

[77] Kitson J.D., McCahon D., Belsham G.J. (1990). Sequence analysis of monoclonal antibody resistant mutants of type O foot and mouth disease virus: Evidence for the involvement of the three surface exposed capsid proteins in four antigenic sites. Virology.

[78] Kleid D.G., Yansura D., Small B., Dowbenko D., Moore D.M., Grubman M.J., McKercher P.D., Morgan D.O., Robertson B.H., Bachrach H.L. (1981). Cloned viral protein vaccine for foot-and-mouth disease: Responses in cattle and swine. Science.

[79] Bittle J.L., Houghten R.A., Alexander H., Shinnick T.M., Sutcliffe J.G., Lerner R.A., Rowlands D.J., Brown F. (1982). Protection against foot-and-mouth disease by immunization with a chemically synthesized peptide predicted from the viral nucleotide sequence. Nature.

[80] Clarke B.E., Brown A.L., Currey K.M., Newton S.E., Rowlands D.J., Carroll A.R. (1987). Potential secondary and tertiary structure in the genomic RNA of foot and mouth disease virus. Nucleic Acids Res..

[81] Zhang Q., Yang Y.Q., Zhang Z.Y., Li L., Yan W.Y., Jiang W.J., Xin A.G., Lei C.X., Zheng Z.X. (2002). Immunogenicity of a recombinant fusion protein of tandem repeat epitopes of foot-and-mouth disease virus type Asia 1 for guinea pigs. Acta Virol..

[82] Taboga O., Tami C., Carrillo E., Núñez J.I., Rodríguez A., Saíz J.C., Blanco E., Valero M.L., Roig X., Camarero J.A. (1997). A large-scale evaluation of peptide vaccines against foot-and-mouth disease: Lack of solid protection in cattle and isolation of escape mutants. J. Virol..

[83] Knowles N.J., Samuel A.R. (2003). Molecular epidemiology of foot-and-mouth disease virus. Virus Res..

[84] Beck E., Forss S., Strebel K., Cattaneo R., Feil G. (1983). Structure of the FMDV translation initiation site and of the structural proteins. Nucleic Acids Res..

[85] Dopazo J., Sobrino F., Palma E.L., Domingo E., Moya A. (1988). Gene encoding capsid protein VP1 of foot-and-mouth disease virus: A quasispecies model of molecular evolution. Proc. Natl. Acad. Sci. USA.

[86] Collen T., Dimarchi R., Doel T.R. (1991). A T cell epitope in VP1 of foot-and-mouth disease virus is immunodominant for vaccinated cattle. J. Immunol..

[87] Rodríguez A., Sáiz J.C., Novella I.S., Andreu D., Sobrino F. (1994). Antigenic specificity of porcine T cell response against foot-and-mouth disease virus structural proteins: Identification of T helper epitopes in VP1. Virology.

[88] Carrillo C., Tulman E.R., Delhon G., Lu Z., Carreno A., Vagnozzi A., Kutish G.F., Rock D.L. (2005). Comparative genomics of foot-and-mouth disease virus. J. Virol..

[89] van Lierop M.J., van Noort J.M., Wagenaar J.P., Rutten V.P., Langeveld J., Meloen R.H., Hensen E.J. (1994). T cell-stimulatory fragments of foot-and-mouth disease virus released by mild treatment with cathepsin D. J. Gen. Virol..

[90] Blanco E., McCullough K., Summerfield A., Fiorini J., Andreu D., Chiva C., Borrás E., Barnett P., Sobrino F. (2000). Interspecies major histocompatibility complex-restricted Th cell epitope on foot-and-mouth disease virus capsid protein VP4. J. Virol..

[91] Gerner W., Denyer M.S., Takamatsu H.H., Wileman T.E., Wiesmüller K.H., Pfaff E., Saalmüller A. (2006). Identification of novel foot-and-mouth disease virus specific T-cell epitopes in c/c and d/d haplotype miniature swine. Virus Res..

[92] Weber C.A., Mehta P.J., Ardito M., Moise L., Martin B., De Groot A.S. (2009). T cell epitope: Friend or foe? Immunogenicity of biologics in context. Adv. Drug Deliv. Rev..

[93] Ganges L., Borrego B., Fernández-Pacheco P., Revilla C., Fernández-Borges N., Domínguez J., Sobrino F., Rodriguez F. (2011). DNA immunization of pigs with foot-and-mouth disease virus minigenes: From partial protection to disease exacerbation. Virus Res..

[94] Blanco E., Garcia-Briones M., Sanz-Parra A., Gomes P., De Oliveira E., Valero M.L., Andreu D., Ley V., Sobrino F. (2001). Identification of T-cell epitopes in nonstructural proteins of foot-and-mouth disease virus. J. Virol..

[95] Blanco E., Guerra B., de la Torre B.G., Defaus S., Dekker A., Andreu D., Sobrino F. (2016). Full protection of swine against foot-and-mouth disease by a bivalent B-cell epitope dendrimer peptide. Antivir. Res..

[96] Cubillos C., de la Torre B.G., Bárcena J., Andreu D., Sobrino F., Blanco E. (2012). Inclusion of a specific T cell epitope increases the protection conferred against foot-and-mouth disease virus in pigs by a linear peptide containing an immunodominant B cell site. Virol. J..

[97] Cubillos C., de la Torre B.G., Jakab A., Clementi G., Borrás E., Bárcena J., Andreu D., Sobrino F., Blanco E. (2008). Enhanced mucosal immunoglobulin A response and solid protection against foot-and-mouth disease virus challenge induced by a novel dendrimeric peptide. J. Virol..

[98] Monsó M., de la Torre B.G., Blanco E., Moreno N., Andreu D. (2013). Influence of conjugation chemistry and B epitope orientation on the immune response of branched peptide antigens. Bioconjug. Chem..

[99] Collen T., Baron J., Childerstone A., Corteyn A., Doel T.R., Flint M., Garcia-Valcarcel M., Parkhouse R.M., Ryan M.D. (1998). Heterotypic recognition of recombinant FMDV proteins by bovine T-cells: The polymerase (P3Dpol) as an immunodominant T-cell immunogen. Virus Res..

[100] Foster M., Cook A., Cedillo L., Parkhouse R.M. (1998). Serological and cellular immune responses to non-structural proteins in animals infected with FMDV. Vet. Q..

[101] García-Briones M.M., Russell G.C., Oliver R.A., Tami C., Taboga O., Carrillo E., Palma E.L., Sobrino F., Glass E.J. (2000). Association of bovine DRB3 alleles with immune response to FMDV peptides and protection against viral challenge. Vaccine.

[102] Cañas-Arranz R., Forner M., Defaus S., Rodríguez-Pulido M., de León P., Torres E., Bustos M.J., Borrego B., Sáiz M., Blanco E. (2020). A bivalent B-cell epitope dendrimer peptide can confer long-lasting immunity in swine against foot-and-mouth disease. Transbound. Emerg. Dis..

[103] Patch J.R., Pedersen L.E., Toka F.N., Moraes M., Grubman M.J., Nielsen M., Jungersen G., Buus S., Golde W.T. (2011). Induction of foot-and-mouth disease virus-specific cytotoxic T cell killing by vaccination. Clin. Vaccine Immunol..

[104] Barfoed A.M., Rodriguez F., Therrien D., Borrego B., Sobrino F., Kamstrup S. (2006). DNA immunization with 2C FMDV non-structural protein reveals the presence of an immunodominant CD8+, CTL epitope for Balb/c mice. Antivir. Res..

[105] Gao F.S., Feng L., Zhang Q., Yan R.Q., Li Y.G., Li X.S. (2013). Immunogenicity of two FMDV nonameric peptides encapsulated in liposomes in mice and the protective efficacy in guinea pigs. PLoS ONE.

[106] Gao F.S., Feng L., Jiang P., Li Z.B., Gao H., Zhai X.X., Zhang Z.H., Hu X. (2018). Expression, purification, crystallization and preliminary X-ray diffraction analysis of swine leukocyte antigen 2 complexed with a CTL epitope AS64 derived from Asia1 serotype of foot-and-mouth disease virus. BMC Vet. Res..

[107] Gao F.S., Zhai X.X., Jiang P., Zhang Q., Gao H., Li Z.B., Han Y., Yang J., Zhang Z.H. (2018). Identification of two novel foot-and-mouth disease virus cytotoxic T lymphocyte epitopes that can bind six SLA-I proteins. Gene.

[108] Pandya M., Rasmussen M., Hansen A., Nielsen M., Buus S., Golde W., Barlow J. (2015). A modern approach for epitope prediction: Identification of foot-and-mouth disease virus peptides binding bovine leukocyte antigen (BoLA) class I molecules. Immunogenetics.

[109] Svitek N., Awino E., Nene V., Steinaa L. (2015). BoLA-6*01301 and BoLA-6*01302, two allelic variants of the A18 haplotype, present the same epitope from the Tp1 antigen of Theileria parva. Vet. Immunol. Immunopathol..

[110] Pedersen L.E., Patch J.R., Kenney M., Glabman R.A., Nielsen M., Jungersen G., Buus S., Golde W.T. (2016). Expanding specificity of class I restricted CD8(+) T cells for viral epitopes following multiple inoculations of swine with a human adenovirus vectored foot-and-mouth disease virus (FMDV) vaccine. Vet. Immunol. Immunopathol..

[111] Pedersen L.E., Harndahl M., Nielsen M., Patch J.R., Jungersen G., Buus S., Golde W.T. (2013). Identification of peptides from foot-and-mouth disease virus structural proteins bound by class I swine leukocyte antigen (SLA) alleles, SLA-1*0401 and SLA-2*0401. Anim. Genet..

[112] Ning S., Wang Z.B., Qi P., Xiao J., Wang X.J. (2020). Crystallization of SLA-2*04:02:02 complexed with a CTL epitope derived from FMDV. Res. Vet. Sci..

[113] Laver W.G., Air G.M., Webster R.G., Smith-Gill S.J. (1990). Epitopes on protein antigens: Misconceptions and realities. Cell.

[114] Mateu M.G., Escarmís C., Domingo E. (1998). Mutational analysis of discontinuous epitopes of foot-and-mouth disease virus using an unprocessed capsid protomer precursor. Virus Res..

[115] Hill T.A., Shepherd N.E., Diness F., Fairlie D.P. (2014). Constraining cyclic peptides to mimic protein structure motifs. Angew. Chem..

[116] Borràs E., Giralt E., Andreu D. (1999). A Rationally Designed Synthetic Peptide Mimic of a Discontinuous Viral Antigenic Site Elicits Neutralizing Antibodies. J. Am. Chem. Soc..

[117] Villén J., Rodríguez-Mias R.A., Núñez J.I., Giralt E., Sobrino F., Andreu D. (2006). Rational dissection of binding surfaces for mimicking of discontinuous antigenic sites. Chem. Biol..

[118] Trabi M., Craik D.J. (2002). Circular proteins--no end in sight. Trends Biochem. Sci..

[119] Valero M.L., Camarero J.A., Haack T., Mateu M.G., Domingo E., Giralt E., Andreu D. (2000). Native-like cyclic peptide models of a viral antigenic site: Finding a balance between rigidity and flexibility. J. Mol. Recognit..

[120] Villén J., de Oliveira E., Núñez J.I., Molina N., Sobrino F., Andreu D. (2004). Towards a multi-site synthetic vaccine to foot-and-mouth disease: Addition of discontinuous site peptide mimic increases the neutralization response in immunized animals. Vaccine.

[121] Villén J., Borràs E., Schaaper W.M., Meloen R.H., Dávila M., Domingo E., Giralt E., Andreu D. (2002). Functional mimicry of a discontinuous antigenic site by a designed synthetic peptide. ChemBioBhem.

[122] Pfaff E., Mussgay M., Böhm H.O., Schulz G.E., Schaller H. (1982). Antibodies against a preselected peptide recognize and neutralize foot and mouth disease virus. EMBO J..

[123] Doel T.R., Doel C.M., Staple R.F., DiMarchi R. (1992). Cross-reactive and serotype-specific antibodies against foot-and-mouth disease virus generated by different regions of the same synthetic peptide. J. Virol..

[124] DiMarchi R., Brooke G., Gale C., Cracknell V., Doel T., Mowat N. (1986). Protection of cattle against foot-and-mouth disease by a synthetic peptide. Science.

[125] Morgan D.O., Moore D.M. (1990). Protection of cattle and swine against foot-and-mouth disease, using biosynthetic peptide vaccines. Am. J. Vet. Res..

[126] Gras-Masse H., Ameisen J.C., Boutillon C., Rouaix F., Bossus M., Deprez B., Neyrinck J.L., Capron A., Tartar A. (1992). Synthetic vaccines and HIV-1 hypervariability: A “mixotope” approach. Pept. Res..

[127] Oliveira E., Jiménez-Clavero M.A., Núñez J.I., Sobrino F., Andreu D. (2005). Analysis of the immune response against mixotope peptide libraries from a main antigenic site of foot-and-mouth disease virus. Vaccine.

[128] Doel T.R., Gale C., Brooke G., DiMarchi R. (1988). Immunization against foot-and-mouth disease with synthetic peptides representing the C-terminal region of VP1. J. Gen. Virol..

[129] Barteling S.J. (1988). Possibilities and limitations of synthetic peptide vaccines. Adv. Biotechnol. Process..

[130] Collen T., Goddeeris B.L., Morrison W. (1994). Foot-and-mouth disease virus (Aphthovirus): Viral T-cell epitopes. Cell-Mediated Immunity in Ruminants.

[131] Glass E.J., Millar P. (1995). Bovine T cells preferentially recognize non-viral spacer epitopes in a putative FMDV vaccinal peptide. Vaccine.

[132] Tami C., Taboga O., Berinstein A., Núñez J.I., Palma E.L., Domingo E., Sobrino F., Carrillo E. (2003). Evidence of the coevolution of antigenicity and host cell tropism of foot-and-mouth disease virus in vivo. J. Virol..

[133] Lee H.B., Piao D.C., Lee J.Y., Choi J.Y., Bok J.D., Cho C.S., Kang S.K., Choi Y.J. (2017). Artificially designed recombinant protein composed of multiple epitopes of foot-and-mouth disease virus as a vaccine candidate. Microb. Cell Factories.

[134] Merrifield R.B., Juvvadi P., Andreu D., Ubach J., Boman A., Boman H.G. (1995). Retro and retroenantio analogs of cecropin-melittin hybrids. Proc. Natl. Acad. Sci. USA.

[135] Briand J.P., Benkirane N., Guichard G., Newman J.F., Van Regenmortel M.H., Brown F., Muller S. (1997). A retro-inverso peptide corresponding to the GH loop of foot-and-mouth disease virus elicits high levels of long-lasting protective neutralizing antibodies. Proc. Natl. Acad. Sci. USA.

[136] Tuchscherer G., Mutter M. (1995). Templates in protein de novo design. J. Biotechnol..

[137] Chen Y.S., Hung Y.C., Lin W.H., Huang G.S. (2010). Assessment of gold nanoparticles as a size-dependent vaccine carrier for enhancing the antibody response against synthetic foot-and-mouth disease virus peptide. Nanotechnology.

[138] Chang C.Y., Huang C.C., Deng M.C., Huang Y.L., Lin Y.J., Liu H.M., Lin Y.L., Wang F.I. (2012). Antigenic mimicking with cysteine-based cyclized peptides reveals a previously unknown antigenic determinant on E2 glycoprotein of classical swine fever virus. Virus Res..

[139] Liu T.Y., Hussein W.M., Jia Z., Ziora Z.M., McMillan N.A., Monteiro M.J., Toth I., Skwarczynski M. (2013). Self-adjuvanting polymer-peptide conjugates as therapeutic vaccine candidates against cervical cancer. Biomacromolecules.

[140] Tam J.P. (1988). Synthetic peptide vaccine design: Synthesis and properties of a high-density multiple antigenic peptide system. Proc. Natl. Acad. Sci. USA.

[141] Tam J.P., Spetzler J.C. (1995). Chemoselective approaches to the preparation of peptide dendrimers and branched artificial proteins using unprotected peptides as building blocks. Biomed. Pept. Proteins Nucleic Acids.

[142] Pessi A., Bianchi E., Chiappinelli L., Bonelli F., Tougne C., Lambert P.H., Del Giudice G. (1991). Multiple antigen peptides (MAPs) as candidate vaccines against malaria. Parassitologia.

[143] Mahajan B., Berzofsky J.A., Boykins R.A., Majam V., Zheng H., Chattopadhyay R., de la Vega P., Moch J.K., Haynes J.D., Belyakov I.M. (2010). Multiple antigen peptide vaccines against Plasmodium falciparum malaria. Infect. Immun..

[144] Heegaard P.M., Boas U., Sorensen N.S. (2010). Dendrimers for vaccine and immunostimulatory uses. A review. Bioconjug. Chem..

[145] Kowalczyk W., de la Torre B.G., Andreu D. (2010). Strategies and limitations in dendrimeric immunogen synthesis. The influenza virus M2e epitope as a case study. Bioconjug. Chem..

[146] Kowalczyk W., Monsó M., de la Torre B.G., Andreu D. (2011). Synthesis of multiple antigenic peptides (MAPs)-strategies and limitations. J. Pept. Sci..

[147] Forner M., Defaus S., Andreu D. (2020). Peptide-Based Multiepitopic Vaccine Platforms via Click Reactions. J. Org. Chem..

[148] Monsó M., Kowalczyk W., Andreu D., de la Torre B.G. (2012). Reverse thioether ligation route to multimeric peptide antigens. Org. Biomol. Chem..

[149] Blanco E., Cubillos C., Moreno N., Bárcena J., de la Torre B.G., Andreu D., Sobrino F. (2013). B epitope multiplicity and B/T epitope orientation influence immunogenicity of foot-and-mouth disease peptide vaccines. Clin. Dev. Immunol..

[150] Soria I., Quattrocchi V., Langellotti C., Gammella M., Digiacomo S., Garcia de la Torre B., Andreu D., Montoya M., Sobrino F., Blanco E. (2017). Dendrimeric peptides can confer protection against foot-and-mouth disease virus in cattle. PLoS ONE.

[151] Soria I., Quattrocchi V., Langellotti C., Pérez-Filgueira M., Pega J., Gnazzo V., Romera S., Schammas J., Bucafusco D., Di Giacomo S. (2018). Immune Response and Partial Protection against Heterologous Foot-and-Mouth Disease Virus Induced by Dendrimer Peptides in Cattle. J. Immunol. Res..

[152] Cañas-Arranz R., Forner M., Defaus S., de León P., Bustos M.J., Torres E., Sobrino F., Andreu D., Blanco E. (2020). A Single Dose of Dendrimer B(2)T Peptide Vaccine Partially Protects Pigs against Foot-and-Mouth Disease Virus Infection. Vaccines.

[153] Defaus S., Forner M., Cañas-Arranz R., de León P., Bustos M.J., Rodríguez-Pulido M., Blanco E., Sobrino F., Andreu D. (2020). Designing Functionally Versatile, Highly Immunogenic Peptide-Based Multiepitopic Vaccines against Foot-and-Mouth Disease Virus. Vaccines.

[154] Cañas-Arranz R., de León P., Forner M., Defaus S., Bustos M.J., Torres E., Andreu D., Blanco E., Sobrino F. (2020). Immunogenicity of a Dendrimer B(2)T Peptide Harboring a T-Cell Epitope From FMDV Non-structural Protein 3D. Front. Vet. Sci..

[155] de León P., Cañas-Arranz R., Defaus S., Torres E., Forner M., Bustos M.J., Revilla C., Dominguez J., Andreu D., Blanco E. (2021). Swine T-Cells and Specific Antibodies Evoked by Peptide Dendrimers Displaying Different FMDV T-Cell Epitopes. Front. Immunol..

[156] de León P., Cañas-Arranz R., Saez Y., Forner M., Defaus S., Cuadra D., Bustos M.J., Torres E., Andreu D., Blanco E. (2020). Association of Porcine Swine Leukocyte Antigen (SLA) Haplotypes with B- and T-Cell Immune Response to Foot-and-Mouth Disease Virus (FMDV) Peptides. Vaccines.

[157] Ji W., Guo Z., Ding N.Z., He C.Q. (2015). Studying classical swine fever virus: Making the best of a bad virus. Virus Res..

[158] Dong X.N., Qi Y., Ying J., Chen X., Chen Y.H. (2006). Candidate peptide-vaccine induced potent protection against CSFV and identified a principal sequential neutralizing determinant on E2. Vaccine.

[159] Lin M., Lin F., Mallory M., Clavijo A. (2000). Deletions of structural glycoprotein E2 of classical swine fever virus strain alfort/187 resolve a linear epitope of monoclonal antibody WH303 and the minimal N-terminal domain essential for binding immunoglobulin G antibodies of a pig hyperimmune serum. J. Virol..

[160] Monsó M., Tarradas J., de la Torre B.G., Sobrino F., Ganges L., Andreu D. (2011). Peptide vaccine candidates against classical swine fever virus: T cell and neutralizing antibody responses of dendrimers displaying E2 and NS2-3 epitopes. J. Pept. Sci..

[161] Tarradas J., Monsó M., Muñoz M., Rosell R., Fraile L., Frías M.T., Domingo M., Andreu D., Sobrino F., Ganges L. (2011). Partial protection against classical swine fever virus elicited by dendrimeric vaccine-candidate peptides in domestic pigs. Vaccine.

[162] Dong X.N., Chen Y.H. (2006). Spying the neutralizing epitopes on E2 N-terminal by candidate epitope-vaccines against classical swine fever virus. Vaccine.

[163] Dong X.N., Chen Y.H. (2006). Candidate peptide-vaccines induced immunity against CSFV and identified sequential neutralizing determinants in antigenic domain A of glycoprotein E2. Vaccine.

[164] Dong X.N., Wei K., Liu Z.Q., Chen Y.H. (2002). Candidate peptide vaccine induced protection against classical swine fever virus. Vaccine.

[165] Liu S., Tu C., Wang C., Yu X., Wu J., Guo S., Shao M., Gong Q., Zhu Q., Kong X. (2006). The protective immune response induced by B cell epitope of classical swine fever virus glycoprotein E2. J. Virol. Methods.

[166] Bohórquez J.A., Defaus S., Muñoz-González S., Perez-Simó M., Rosell R., Fraile L., Sobrino F., Andreu D., Ganges L. (2017). A bivalent dendrimeric peptide bearing a T-cell epitope from foot-and-mouth disease virus protein 3A improves humoral response against classical swine fever virus. Virus Res..

[167] Li G.X., Zhou Y.J., Yu H., Li L., Wang Y.X., Tong W., Hou J.W., Xu Y.Z., Zhu J.P., Xu A.T. (2012). A novel dendrimeric peptide induces high level neutralizing antibodies against classical swine fever virus in rabbits. Vet. Microbiol..

[168] Sadler K., Tam J.P. (2002). Peptide dendrimers: Applications and synthesis. J. Biotechnol..

[169] Purcell A.W., McCluskey J., Rossjohn J. (2007). More than one reason to rethink the use of peptides in vaccine design. Nat. Rev. Drug Discov..

[170] Li W., Joshi M.D., Singhania S., Ramsey K.H., Murthy A.K. (2014). Peptide Vaccine: Progress and Challenges. Vaccines.

[171] Sabatino D. (2020). Medicinal Chemistry and Methodological Advances in the Development of Peptide-Based Vaccines. J. Med. Chem..

[172] Combadière B., Beaujean M., Chaudesaigues C., Vieillard V. (2019). Peptide-Based Vaccination for Antibody Responses Against HIV. Vaccines.

[173] Lamonaca V., Missale G., Urbani S., Pilli M., Boni C., Mori C., Sette A., Massari M., Southwood S., Bertoni R. (1999). Conserved hepatitis C virus sequences are highly immunogenic for CD4(+) T cells: Implications for vaccine development. Hepatology.

